# Postoperative Pain, an Unmet Problem in Day or Overnight Italian Surgery Patients: A Prospective Study

**DOI:** 10.1155/2016/6104383

**Published:** 2016-12-28

**Authors:** Sara Campagna, Maria Delfina Antonielli D'Oulx, Rosetta Paradiso, Laura Perretta, Silvia Re Viglietti, Paola Berchialla, Valerio Dimonte

**Affiliations:** ^1^School of Nursing, Department of Clinical and Biological Science, University of Torino, Via San Giacomo 2, Beinasco, 10092 Torino, Italy; ^2^General Surgery, Hospital Evangelico Valdese, Via Pellico 19, 10135 Torino, Italy; ^3^General Surgery, Hospital Ospedale Umberto I, Via Magellano N.1, 10128 Torino, Italy; ^4^Department of Clinical and Biological Science, University of Torino, Via Santena 5bis, 10126 Torino, Italy; ^5^Department of Public Health and Paediatrics, University of Torino, Via Santena 5bis, 10126 Turin, Italy

## Abstract

*Background.* Because of economic reasons, day surgery rates have steadily increased in many countries and the trend is to perform around 70% of all surgical procedures as day surgery. Literature shows that postoperative pain treatment remains unfulfilled in several fields such as orthopedic and general surgery patients. In Italy, the day surgery program is not yet under governmental authority and is managed regionally by local practices.* Aim*. To investigate the trends in pain intensity and its relation to type of surgeries and pain therapy protocols, in postoperative patients, discharged from three different Ambulatory Surgeries located in North West Italy (Piedmont region).* Method*. The present study enrolled 276 patients who undergone different surgical procedures in ambulatory regimen. Patients recorded postoperative pain score twice a day, compliance with prescribed drugs, and pain related reasons for contacting the hospital. Monitoring lasted for 7 days.* Results*. At discharge, 72% of patients were under weak opioids, 12% interrupted the treatment due to side effects, 17% of patients required extra drugs, and 15% contacted the hospital reporting pain problems. About 50% of patients experienced moderate pain during the first day after surgery. Results from our study show that most of the patients experienced avoidable pain after discharge.

## 1. Introduction

To enable rapid discharge and more efficient use of beds, many surgeries are performed in ambulatory regimens; in the near future, advances in techniques and methods will allow 50 to 75% of all surgical conditions to be treated on a day basis [1].

Italy is currently performing about 29% of all surgery in ambulatory regimen and since only larger hospitals have dedicated units, it is mainly carried out in hospital facilities [1].

Day surgery requires patient safety and satisfaction with care: good analgesia is said to be one of the key factors for successful ambulatory surgery [2]. Postoperative pain management, however, remains challenging. Generally, pain decreases over time but may persist for days or even months [3] and it can influence the patient's quality of life for up to 6 months after discharge [4, 5].

The level of pain 48 hours after surgery is a good predictor for returning to work; the more severe reported pain at 48 hours, the greater risk for the patient to resume normal activity within one week [6]. Even mild pain (NRS 1–3/10) may negatively interfere with daily living, work, and movement [6], and it is responsible for inactivity in 54% of patients [7].

Postoperative pain assessment and treatment are also clinically important since pain is related to increased vascular resistance and cardiac workload, particularly in the elderly [8]. Furthermore, untreated or undertreated pain may persist and become chronic in 10 to 50% of the patients [9].

Before the year 2010, studies reported moderate-to-severe pain scores in more than 30% of day hospital patients 24 hours after surgery [7, 10], and the overall incidence of pain after discharge was approximately 57% [11, 12].

Recently, studies reported moderate-to-severe pain after hospital discharge (general, orthopedic, and urological procedures) in 50% of patients after 48 hours and in 40% still after one week; moderate-to-severe pain can persist in 20 to 30% of patients after 3 to 4 months [6, 13].

One of the strongest unique predictors of satisfaction with treatment is patients' belief that their pain has been thoroughly evaluated [14] but studies show that postoperative pain is frequently poorly assessed and poorly documented [15].

A large survey showed that even anesthesiologists believe that postoperative pain in day surgery still seems to be a problem and that almost half of the ambulatory patients experience high pain scores at home [16].

Several studies carried out in countries with high rates of day surgery activities and standardized procedures have shown that day surgery procedures vary greatly across European countries [17] and the use of analgesics at home is influenced by patients' knowledge and attitude [18].

The recent large POPSI and POPSI-2 studies (Postoperative Pain Surveys in Italy from 2006 and 2012) have indicated that the management of postoperative pain in Italy is still suboptimal and below European standards [19, 20].

Italian care settings, perioperative therapies, and home prescriptions after day surgery vary among different hospitals. In 2012 about 55% of Italian anesthesiologists stated that they used hospital protocols and 13% did not use any protocols; this was more likely to occur in Northern Regions [20].

The aim of this paper is to investigate the incidence and the course of postoperative pain one week after ambulatory surgery and patients home habits related to prescribed therapy. To our knowledge there are no Italian studies on postoperative pain after day surgery and patients' behavior towards drugs after discharge.

## 2. Methods

A prospective 3-month observational study was carried out in a convenience sample of 3 different day surgery units in Piedmont Hospitals (North West Italy) in 2012. All patients over 18 years of age, being able to fluently speak in Italian and undergoing surgery in ambulatory regimen, were included.

Demographic data, type of surgery, anesthesia modality, prescribed drugs and other treatments, pain killers in the first 24 hours, and treatment compliance were collected. Survey started on day of surgery (day 1), ending on day seven. Patients received a diary (see Supplemental Material available online at http://dx.doi.org/10.1155/2016/6104383) at discharge in which they recorded twice daily (8 am and 8 pm) for 7 days the pain experienced on a numeric scale (NRS) from 0 (no pain) to 10 (maximum pain). Patients were asked to report compliance with prescribed drugs and reasons for stopping drug use, any other pain treatment, and reasons for contacting general practitioners or hospital staff.

Since procedures are influenced by local practices, data from clinical records were collected by 3 research nurses and the information concerning diary patients' compilation was provided by ward nurses.

In the three examined settings, perioperative therapy and home prescriptions after day surgery vary and they are prescribed on the basis of hospital protocols or on the basis of anesthetists or surgeons' experiences and preferences. Due to the heterogeneity of these hospitals, drugs were coded for data analysis on the WHO ladder as follows [21, 22]: (i)* no opioids* (500/1000 mg of acetaminophen alone or with 30 mg of ketorolac every 6–8 hours); (ii)* weak opioids* (500 mg of acetaminophen with 30 mg of codeine every 6–8 hours or 50 mg of tramadol in a 250 mL saline solution drip at 20 mL/h); and (iii)* strong opioids* (10 mg of morphine in a 250 mL saline solution drip at 30 mL/h or 0.6 mg of buprenorphine in a 500 mL saline solution drip at 20–50 mL/h). Intravenous infusions were positioned at the exit of the operating room and maintained up to the time of discharge.

Analgesic protocols consisted of oral tablets; agents, doses, and administration frequency were prescribed at discharge by ward surgeons. For the descriptive analysis drugs were coded on the WHO ladder as follows [21, 22]: (i) no opioids (600 mg of ibuprofen or 1 g of acetaminophen) on a regular basis or PRN (pro re nata) and (ii) weak* opioids* (500 mg of acetaminophen with 30 mg of codeine) on a regular basis or PRN.

### 2.1. Statistical Methods

Continuous data were analyzed as means (standard deviations) or median (range) according to the type of distribution. Discrete data were analyzed as counts and percentages. A threshold of NRS ≥ 4 was used to discriminate between mild and moderate-to-severe pain of postoperative average pain intensity [8]. NRS scores were categorized as follows: 0 = no pain; 1–3 = mild; 4–6 = moderate; and 7–10 = severe.

Therapy at discharge and type of anesthesia were analyzed using mixed-effects regression models to identify associations between NRS scores over time and type of surgery. A compound symmetry structure for repeated measurements of the same patients (at 24 and 48 hours and 7 days) corresponding to a constant correlation over time resulted in the best model fit based on Akaike Information Criterion (AIC) values. Adjustment for sex and age was considered.

All analyses were performed using R (The R Project for Statistical Computing) version 2.15.

### 2.2. Consensus and Privacy

Institutional Review Board of each hospital approved the study. Patients were identified only by the nurses on duty, and data collection forms were stored in a locked area accessible only by authorized personnel until data entry was completed. All patients signed an informed consent form.

## 3. Results

A total of 276 patients were recruited (Table 1) with a mean age of 56.1 (±14.2) years; 53% underwent general surgery, and 47% underwent orthopedic surgery. Forty-five patients discontinued the compilation of the diary until the second postoperative day and 5 patients discontinued on the fourth postoperative day, because the pain disappeared.

### 3.1. Pain Therapy

Around 84% of patients received regional anesthesia and 16% general anesthesia. Analgesia techniques (Table 2) varied among orthopedic (more troncular analgesia) and general surgical patients (more general analgesia) and the results are statistically significant.

Pain therapy after surgery in the recovery room included intravenous nonopioids (48%), weak opioids (39%), and strong opioids (13%). During the postoperative hours (Table 3), 59% of orthopedic patients were prescribed a nonopioid (acetaminophen alone or plus ketorolac) and 11% strong opioids (morphine or buprenorphine). While 47% of general surgical patients received weak opioids (acetaminophen + codeine or tramadol) and 13% strong opioids.

At discharge (Table 4), prescribed therapies have been heterogeneous for type of intervention. Overall, about 70% of orthopedic and general surgical patients were prescribed weak opioids. Of the patients who were prescribed weak opioids at discharge, 12% patients interrupted the treatment because of side effects, particularly nausea, vomiting, and gastric pain.

### 3.2. Pain Intensity

**Figure 1:**
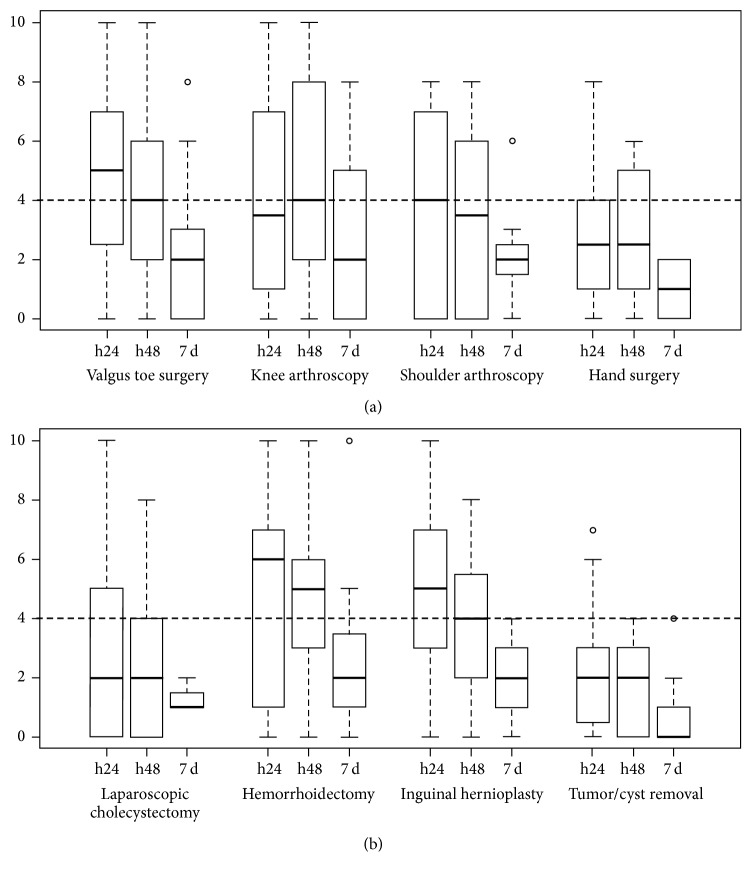
Box plot of pain scores for orthopedic (a) and general (b) surgeries.

Postoperative pain varied across surgeries (Figure 1). Moderate or severe pain was experienced by 51% patients 24 hours after surgery (23% and 28.6%, resp.), and 38% patients experienced moderate or severe pain 48 hours after surgery (11% and 27.5%, resp.). The most painful surgeries, as indicated by the highest proportion of patients with moderate-to-severe pain after 24 hours, were valgus toe surgery (70%), hemorrhoidectomy (68%), inguinal hernioplasty (67%), and knee and shoulder arthroscopy (over 50%). Nine percent experienced moderate-to-severe pain one week after surgery. Most patients still having pain after one week had also experienced pain during the first 48 hours.

Although numbers were limited, about 30% of patients who were prescribed weak opioids on a regular basis and about 70% of patients who were prescribed weak opioids drugs PRN experienced moderate-to-severe pain at 24 and 48 hours (Table 4).

Regression analysis (Table 5) shows a mean reduction of pain over time equal to 2.67 (orthopedic surgery, 0.89 point reduction in pain every 12 hours, *p* < 0.001) and 2.91 (general surgery, 0.97 points reduction in pain every 12 hours, *p* < 0.001). Patients who took extra drugs, at home, have significantly higher level of pain (a mean of 3.42 for orthopedic surgery and 2.86 points for general surgery). Pain is decreased by a further 0.89 points (orthopedic surgery) and 0.77 points (general surgery) every 12 hours, *p* < 0.01. Hand surgery was significantly less painful than valgus toe surgery as well as tumor/cyst removal as compared to laparoscopic cholecystectomy. The hospital effect is negligible for both orthopedic and general surgery.

### 3.3. Patients' Pain Relief Strategies at Home

Overall 11% of patients took no analgesics at home; about 76% of them had no or only mild pain, but the other patients experienced moderate-to-severe pain.

About 15% of patients contacted health care professionals because of pain: the general practitioner, the emergency department, or the surgical ward. Eight of these patients had mild pain, 16 patients had moderate pain, and 13 patients had severe pain.

Forty-six patients (16.7%) took extra analgesics: half of them underwent valgus toe surgeries and knee arthroscopies and half of these patients underwent herniorrhaphies, hemorrhoidectomies, and exercises.

Nonpharmacological interventions for pain control at home were reported by 17.8% of patients; 83% used ice bags, and the other used massage.

## 4. Discussion

Early discharge is increasingly being adopted and demands a rapid recovery and low incidence and intensity of surgery and anesthesia related side effects to guarantee feasibility of self-care at home.

The present study demonstrates, in accordance with the literature, that postoperative pain is an important factor complicating recovery and discharge of patients after day surgery procedures.

Prevalence of pain after orthopedic and general surgeries is consistent with recent studies in other countries [6, 7, 11, 12, 18, 23–25].

After one week, patients who had undergone hemorrhoidectomy and valgus toe surgery still experienced moderate-to-severe pain. This is an unmanaged problem that even affects long-term outcomes. High levels of pain 4 days after surgery were associated with a worse quality of life at 6 months [5]. Viñoles et al. [10] reported that between 40 and 56% of hemorrhoidectomy, inguinal hernioplasty and valgus toe surgery patients had a high level of ambulatory surgical incapacity and required more accurate multimodal analgesic strategies.

Pain management strategies show some differences among patients that could be explained with the different prescriptions protocols adopted by each surgeon. The literature shows different sight on pain intensity and duration related to surgical procedure. A recent study demonstrated that inpatients undergoing minor surgeries reported high pain score but often were ignored or not taken seriously, so that analgesic administration was delayed and/or insufficient [26].

The general strategy for perioperative pain control is fundamentally founded on the use of systemic analgesics, by intravenous or oral route, with few targeted to the individual patient's needs. Many anesthesiologists preferred this approach because it is easy to use and can be managed by nurses as well as being considered cheaper [19].

Although not recommended, PRN drugs are prescribed as the primary pain therapy for all surgeries except for laparoscopic surgery [13, 27].

Several alternatives that seem to provide better pain relief, such as patient controlled regional analgesia (PCA) and regional blocks with catheters in situ, are reported in guidelines [1]. However, in Italy, access to PCA pump equipment and the lack of primary care are the main impediments to their use [20].

Individual patient variability is a well-known topic: for the same surgical intervention and painkillers administered, patients report different levels of pain and response. This problem seems to be unsolvable but surely manageable if the patient could be monitored more closely and fastened follow-up after discharge [28].

Only 50% of all Italian hospitals have an active APS (Acute Pain Service), and improving postoperative pain control is affected by organizational and cultural barriers [20]. No hospital had an active APS at the time of present study. Currently there is a lack of human and technological resources and a misconception that pain control is needed for only few hours or days [29].

Patient behaviors at home confirm that pain is a multifactorial experience not just a symptom, and patient tends to manage it in “his/her own way.” Some patients take analgesics only when “really needed” while others take nothing “even when it is needed.” This suggests that informing a patient does not mean involving a patient and that preoperative and postoperative care should be improved [30].

Furthermore, clinical information provided by physicians and nurses before surgery during few minutes session often overload patients with details which are difficult to process and retain [31].

We observed that ice bags were used only by 20% of patients while they were routinely recommended after each orthopedic procedure, and the reasons for lack of compliance should be explored. Other studies demonstrated that only a limited number of patients (3% of shoulder and 4% of hand surgery patients) used cold treatment [23]. Patients might not consider cold treatment as being effective to relieve pain.

Despite the data being collected in 2012, the anesthesiology procedures and pain treatment strategies have remained substantially the same, so they are still up-to-date.

There are some limitations in the study. First, it was carried out on a convenience sample without a formal sample calculation because of the explorative nature of the study. On the basis of a posterior power analysis, the sample size of 276 patients allows for a power of 79% to assess a difference of 1 point in pain score over time, considering a standard deviation of 3 points and a I-type error of 5% on the basis of a paired *t*-test.

In the study we gave priority to the simplicity of compilation tools and we did not consider other important outcomes factors like pain during ambulation. This is probably one of the most important pain factors for ambulatory patients since they have to cope with pain during movement at home with limited additional aid.

We have not investigated the surgical technique because of hospital organizational settings.

Several patients in this study received a local or spinal anesthesia, so pain intensities collected in this cohort study may differ from those obtained with other patients group.

Awareness of postoperative pain intensity after day surgery procedures may contribute to improving postoperative care by implementation of specific pain treatment protocols and home follow-up.

## 5. Conclusion

One of the key elements of a safe discharge home of day surgery patients is good knowledge of the postoperative course, including pain management. Our data indicate that pain varied widely across patients and surgeries, emphasizing the need for personalized pain treatment. Preoperative screening for patients who are at risk of postoperative pain would enable personalized pain control interventions and closer patient follow-up allowing management of tailored therapy based on the real need. Management of pain and side effects of painkillers should be a priority for quality improvements of day surgery program.

## Supplementary Material

The data collection form was filled in by nurses while the diary was completed by patients from the time of surgery until the end of the seventh day after surgery and returned to the follow-up visit.

## Figures and Tables

**Table 1:** Sample description.

	*N* (%)
Age, mean (SD)	56.1 (±14.2)
Males	126 (46)
*Orthopedic surgery*	
Valgus toe surgery	60 (21.7)
Knee arthroscopy	35 (12.7)
Hand surgery	22 (8.0)
Shoulder arthroscopy	11 (4.0)
*General surgery*	
Inguinal hernioplasty	57 (20.7)
Tumor/cyst removal	53 (19.2)
Hemorrhoidectomy	25 (9.1)
Laparoscopic cholecystectomy	13 (4.6)
*Anesthesia*	
Spinal	88 (31.9)
Local	72 (26.1)
Nerve/block	72 (26.1)
General	44 (15.9)
*Postoperative analgesia in recovery room*	
Nonopioids	44 (47.8)
Weak opioids	108 (39.1)
Strong opioids	35 (12.7)
No analgesia	1 (0.4)
*Pain therapy at home*	
Weak opioids regular basis	172 (62.3)
Nonopioids regular basis	52 (18.8)
Weak opioids PRN	28 (10.1)
Nonopioids PRN	20 (7.2)
No treatment	4 (1.6)

**Table 2:** Anesthesia by type of surgery.

	Orthopedic surgery				Total^*∗*^	General surgery				Total^*∗*^
	Valgus toe	Knee arthroscopy	Shoulder arthroscopy	Hand surgery		laparoscopic cholecystectomy	Hemorrhoidectomy	Inguinal hernioplasty	Tumor/cyst removal	
	60	35	11	22	*128*	13	25	57	53	*148*
Anesthesia										
Local	7 (11.7)	7 (20.0)	1 (9.1)	3 (13.6)	*18 (14.1)*	0 (0.0)	5 (20.0)	9 (15.8)	40 (75.5)	*54 (36.5)*
General	0 (0.0)	4 (11.4)	4 (36.4)	0 (0.0)	*8 (6.2)*	13 (100)	2 (8.0)	18 (31.6)	3 (5.7)	*36 (24.3)*
Spinal	8 (13.3)	24 (68.6)	0 (0.0)	2 (9.1)	*34 (26.6)*	0 (0.0)	18 (72.0)	30 (52.6)	6 (11.3)	*54 (36.5)*
Troncular	45 (75.0)	0 (0.0)	6 (54.5)	17 (77.3)	*68 (53.1)*	0 (0.0)	0 (0.0)	0 (0.0)	4 (7.5)	*4 (2.7)*

^*∗*^ *p* < 0.001 (Fisher's test) significant differences between orthopedic and general surgery (test performed between overall orthopedic surgery and overall general surgery without distinguishing the specific surgery intervention).

**Table 3:** Postoperative analgesia in recovery room by type of surgery.

Surgery	Nonopioid	Weak opioid	Strong opioid	Total
*Orthopedic surgery*				
Valgus toe surgery	38	15	7	60
Shoulder arthroscopy	2	5	4	11
Knee arthroscopy	24	8	3	35
Hand surgery	11	10	1	22
*Total N (%)*	*75 (59)*	*38 (30)*	*15 (11)*	*128*
*General surgery*				
Hemorrhoidectomy (25)	12	8	5	25
Inguinal hernioplasty (57)	31	19	7	57
Laparoscopic cholecystectomy (13)	0	5	8	13
Tumor/cyst removal (53)	14	38	0	52^*∗*^
*Total N (%)*	*57 (39)*	*70 (47)*	*20 (13)*	*147*
*Orthopedic and general surgery N (%)*	*132 (48)*	*108 (39)*	*35 (13)*	*1 (1)*

^*∗*^One patient had no therapy.

**Table 4:** Discharge therapies by type of surgery and administration time.

Therapy at discharge	Nonopioids regular basis	Weak opioids regular basis	No opioids PRN^*∗*^	Weak opioids PRN^*∗*^	No therapy
*Orthopedic surgery*					
Valgus toe surgery (60)	0	58	2	0	0
Shoulder arthroscopy (11)	3	7	1	0	0
Knee arthroscopy (35)	16	13	5	0	1
Laparoscopic cholecystectomy (22)	4	12	5	0	1
*Total N (%) *	*23 (18)*	*90 (70)*	*13 (10)*	*0 (0)*	*2 (2)*

*General surgery*					
Hemorrhoidectomy (25)	9	7	1	7	1
Inguinal hernioplasty (57)	12	24	0	21	0
Laparoscopic cholecystectomy (13)	3	10	0	0	0
Tumor/cyst removal (53)	5	41	6	0	1
*Total N (%)*	*29 (20)*	*82 (55)*	*7 (5)*	*28 (19)*	*2 (1)*

*Pain at 24 hours*					
No pain/mild pain	13/18	30/77	4/10	0/8	0/2
Moderate/severe pain	13/8 (40.3)	47/16 (37)	2/4	12/8 (71.4)	1/0
*Median pain (range)*	*4 (0–10)*	*3 (0–10)*	*3 (0–8)*	*6,5 (1–9)*	*1 (1–6)*

*Pain at 48 hours*					
No/mild pain	10/21	26/77	2/8	0/8	1/2
Moderate/severe pain	11/5 (34)	33/7(28)	3/2	13/3 (66.6)	0/0
*Median pain (range)*	*4 (0–10)*	*3 (0–10)*	*3 (0–8)*	*5 (1–8)*	*2 (0–4)*

^*∗*^PRN = pro re nata (as needed).

**Table:** (a) Orthopedic surgery

Variable	Coeff	SE	*p* value
Time	*−0.89*	*0.15*	*<0.001*
Extra drugs use	*3.42*	*0.77*	*<0.001*
Surgery (ref: valgus toe)			
Knee arthroscopy	−0.73	0.69	0.30
Shoulder arthroscopy	−1.14	0.80	0.15
Hand surgery	*−1.35*	*0.58*	*0.02*
Anesthesia (ref: general)			
Local	0.32	1.06	0.76
Spinal	0.45	0.99	0.65
Troncular	0.99	0.98	0.31
Males	0.29	0.47	0.53
Age	−0.04	0.02	0.01
Time × extra drugs use	*−0.89*	*0.31*	*<0.01*

Hospital effect < 0.1% (only two hospitals).

**Table:** (b) General surgery

Variable	Coeff	SE	*p* value
Time	*−0.97*	*0.11*	*<0.001*
Extra drugs use	*2.86*	*0.61*	*<0.001*
Surgery (ref: laparoscopic cholecystectomy)			
Hemorrhoidectomy	0.94	0.75	0.21
Inguinal hernioplasty	1.01	0.70	0.15
Tumor/cyst removal	*−1.53*	*0.77*	*0.05*
Anesthesia (ref: general)			
Local	0.75	0.55	0.19
Spinal	1.17	0.52	0.03
Troncular	1.64	1.13	0.15
Males	−0.24	0.39	0.54
Age	−0.01	0.01	0.26
Time × extra drug use	*−0.77*	*0.24*	*<0.01*

Hospital effect 2.29%.
