# Supplementary material for: Postoperative Pain, an Unmet Problem in Day or Overnight Italian Surgery Patients: A Prospective Study
Source: Pain Res Manag. 2016 Dec 28;2016:6104383. doi: 10.1155/2016/6104383 (PMC5225384; doi:10.1155/2016/6104383)
Supplement: Supplementary file 1 — The data collection form was filled in by nurses while the diary was completed by patients from the time of surgery until the end of the seventh day after surgery and returned to the follow-up visit. [file 6104383.f1.pdf]

DATA COLLECTION

CC NUMBER: ____________________

SURGICAL INTERVENCTION DATE: ______________________ TELEPHONE CALL DATE: _______________

TYPE OF SURGICAL INTERVENCTION:

____

TYPE OF ANESTHESIA:

- Local
- Local + Sedation
- Nerve Block
- Spinal
- Narcosis
- Other____

SEX: M F AGE: _______

ANALGESIC THERAPY AFTER SURGERY (prescribed for the first 24 h):

- Tachidol every___h
- Perfalgan every___h
- Toradol every ___h
- Orudis every ___h
- Oxycontin ___mg every___h
- Other____

ANALGESIC THERAPY AT DISCHARGE:

- Tachidol every___h
- Toradol if pain> 4
- Other____

1) PAIN 6 HOURS AFTER DISCHARGE:

2) PAIN 12 HOURS AFTER DISCHARGE:

3) PAIN 24 HOURS AFTER DISCHARGE:

4) OTHER ASSUMED DRUG PAINKILLERS TO THOSE REQUIRED ON DISCHARGE LETTER:

IF YES, WHAT?

5) HAVE YOU CONTACTED ANY DOCTOR OR HEALTH SERVICE TO MANAGE PAIN?

IF YES, WHO?

DIARY

	Discharge		Day 1		Day 2		Day 3		Day 4		Day 5		Day 6		Day 7	
	h 16	h 22	h 8	h 20	h 8	h 20	h 8	h 8	h 8	h 20	h 8	h 20	h 8	h 20	h 8	h 20
My pain level from 0 to 10																
Did you take painkillers? (yes/no)																
Did you take any other painkiller? (yes/no)																
Did you contact your general practitioner? (yes/no)																
Did you contact your hospital? (yes/no)																
Did you go to Emergency Department? (yes/no)																
Other pain relief strategies (yes/no)																

If you have taken drugs other than prescribed, what you took?

____

If you have used other ways to relief pain, what they did? (Eg. Ice on the operated area, massages, natural remedies ..) They gave you relief? (Yes or no)

____

Was there any symptom / problem that forced you to stop painkillers? (Eg. Nausea, vomiting ...). Describe it, please.

____
